# Genetic Aspects of Susceptibility to Mercury Toxicity: An Overview

**DOI:** 10.3390/ijerph14010093

**Published:** 2017-01-18

**Authors:** Virginia Andreoli, Francesca Sprovieri

**Affiliations:** Institute of Atmospheric Pollution Research, National Research Council of Italy, Division of Rende, Rende 87036, Italy; f.sprovieri@iia.cnr.it

**Keywords:** mercury, toxicokinetics, human health, risk assessment, children exposure, environmental genetics, DNA variants, biomarkers of susceptibility

## Abstract

Human exposure to mercury is still a major public health concern. In this context, children have a higher susceptibility to adverse neurological mercury effects, compared to adults with similar exposures. Moreover, there exists a marked variability of personal response to detrimental mercury action, in particular among population groups with significant mercury exposure. New scientific evidence on genetic backgrounds has raised the issue of whether candidate susceptibility genes can make certain individuals more or less vulnerable to mercury toxicity. In this review, the aim is to evaluate a new genetic dimension and its involvement in mercury risk assessment, focusing on the important role played by relevant polymorphisms, located in attractive gene targets for mercury toxicity. Existing original articles on epidemiologic research which report a direct link between the genetic basis of personal vulnerability and different mercury repercussions on human health will be reviewed. Based on this evidence, a careful evaluation of the significant markers of susceptibility will be suggested, in order to obtain a powerful positive “feedback” to improve the quality of life. Large consortia of studies with clear phenotypic assessments will help clarify the “window of susceptibility” in the human health risks due to mercury exposure.

## 1. Introduction

Mercury (Hg) is a global pollutant and well-known neurotoxin that has raised great fear in the international scientific community, due to a variety of significant and documented adverse effects on human health and the environment throughout the world [1]. Despite being a well-documented systemic toxicant, an understanding of all the molecular mechanisms underlying the damage induced by Hg is still elusive.

The need to further reduce Hg emissions, as well as to develop preventive strategies in relation to Hg risk assessment and management makes the situation even more challenging, especially for those individuals most susceptible to the effects of Hg exposure, such as children and adolescents [1]. The two categories are highly sensitive to the neurotoxic Hg effects, displaying extreme variability in mainly neurological and neurobehavioural outcomes throughout subsequent life stages [2].

The pathological impact of Hg on humans and other organisms is widely proven, and the overall picture is quite complex. Human exposure may occur chronically through a variety of pathways in the world population, including industrial processes, occupational and household uses, dental amalgams, Hg-containing vaccines, consumption of contaminated fish and marine mammals, and many others [2]. To date, two main types of risk for human health have been detected: a direct one, related to the inhalation of gaseous Hg, with several pathophysiological impacts, and collateral risks, related to differences between Hg species.

In the environment, Hg is present in various physical and chemical forms, which have different transport and deposition characteristics and impacts on ecosystems. The speciation of atmospheric Hg is important in order to understand the fate and impact of this pollutant on the environment. Gaseous elemental Hg (Hg^0^) is normally the most abundant form of Hg in the atmosphere (>98%) whereas oxidized Hg compounds are normally found at much lower concentrations (in the pgm^−3^ range) in the air. 

Due to their chemical-physical characteristics, each Hg form differs in terms of environmental behavior, potential interaction with biological processes, toxicokinetics, and clinical significance resulting from the various chemical structures. Therefore, both inorganic and organic Hg compounds (the latter generally found as methyl-Hg, MeHg) give rise to the intricate biogeochemical cycle of Hg [3].

Although all Hg forms have adverse effects on human health at high doses, the evidence that exposure to very low Hg doses may potentially led to significant consequences for humans is still open to wide interpretation. In this respect, the biological monitoring, or biomonitoring, may allow the evaluation of Hg levels in people’s body, providing indirectly information on human health risk among subjects exposed to Hg. 

Therefore, the negative Hg impact on humans is difficult to evaluate in terms of health perspectives, because it offers a multifaceted general picture where chronically exposed people due to lifestyles, cultures, socio-demographic characteristics, and working activities still play a leading role [4].

To aggravate such a delicate situation, Hg toxicity and associated health effects can vary remarkably at an individual level, depending not only on its chemical speciation, concentrations, and time of exposure, but also on the individual susceptibility to Hg hazards. In fact, not all individuals respond similarly under the same Hg exposure conditions, and they often display significant individual differences in Hg accumulation, distribution and elimination. Consequently, it is hard to predict how much someone may be influenced by a given Hg concentration in his body, and establish the highest level considered tolerable for human Hg exposure.

In the light of the above remarks, recent epidemiological studies have attempted to investigate those key factors capable of modifying a personal response to Hg exposure, but with sometimes-conflicting results [5,6,7,8,9,10]. However, the state of the art of currently scientific evidence suggests the potential involvement of individual genetic makeup in mediating human sensitivity to Hg exposure. In this context, genetic determinants within candidate genes would be able to intervene as expression-targets to Hg stimuli, based on their predisposing or protective functions, by acting partially on the inter-individual variation in biomarker levels and health effects [11,12]. 

In the future, it will be necessary an ever-increasing identification of functional polymorphisms as genetic susceptibility factors to Hg-associated health outcomes, to perform a very careful human risk assessment for children and adults. 

In this perspective, the overall goal of this review is to summarize evidence on gene classes of interest and their corresponding genetic polymorphisms, so far evaluated as emerging susceptibility loci to adverse Hg outcomes, focusing on the potential role played by the gene-Hg interactions in this context. 

It is hoped that a better understanding of this new genetic dimension may have significant implications for public health and prevention initiatives, providing important insights useful to the implementation of targeted measures against the toxic effects of Hg.

## 2. Mechanisms of Toxicity

The toxic effects of Hg for several years were mainly associated with the development of neuropathological conditions. Actually, its high toxic potential derives from the same processes (absorption, distribution, metabolism, excretion; ADME) that characterize the toxicokinetics of any heavy metal: it is transported, distributed, and excreted but can sometimes be metabolized [13]. 

In addition, there is some interconversion “in vivo” between the various forms of Hg. In the complex, its toxicity arises from several strands to assess the global risks of human exposures.

Elemental Hg (Hg^0^) presents a very peculiar behavior; it is the only metal found in a liquid state under ambient conditions [14]. In its liquid form, Hg^0^ is poorly absorbed and presents little health risk. Owing to its very low vapor pressure, Hg^0^ readily vaporizes at room temperature and, as such, can be readily absorbed via the lungs, following which it distributes predominantly to the kidneys and the central nervous system (CNS). Consequently, it is a potent, toxic biohazard at room temperatures.

Therefore, Hg^0^ distributes throughout the body, as it easily passes through most cell membranes including the blood-brain barrier (BBB) and the placenta. However, long-term exposure to Hg vapor primarily leads to CNS defects.

Once in the bloodstream, Hg^0^ is oxidized to its mercuric ion (Hg^2+^) by the catalase enzyme. Moreover, since the ions do not cross BBB very well, this process limits Hg absorption although not so quickly as to prevent considerable uptake by CNS, while still in the metallic form.

Intracellularly, Hg^2+^ is able to react with molecules or structures (e.g., enzymes, glutathione, tubulin, ion channels, or transporters), inhibiting their activities and interfering with normal cellular functions and detoxification processes [14]. 

The urine and feces are the main excretory pathways of metallic and inorganic Hg in humans. Urinary Hg originates mainly from Hg in kidney tissue, the main site of Hg mercuric toxicity. Urinary excretion can reflect this form of Hg, especially when a significant source of elemental Hg exposure exists. In the complex, urine is the commonly used biological marker, as it represents the exposure to inorganic Hg [13].

MeHg represents one of the best-known neurotoxins. Of course, this critically hazardous and ubiquitous Hg form in marine ecosystems [15] is the prime interest of both epidemiological and experimental research, among the organic Hg compounds. Usually produced from biological sources by microbial action, MeHg enters the aquatic food chain, where it undergoes a remarkable bioaccumulation process in muscle tissues of fish, particularly of long-lived predatory species, as well as in freshwater and marine systems [16]. 

Accordingly, human exposure occurs from consumption of food derived from aquatic animals with bioaccumulated MeHg, especially in populations living close to oceans, lakes and rivers [15]. However, this very harmful form can cause nuanced clinical pictures against a backdrop of widespread environmental contamination, with severe neurological damage to humans and wildlife [17]. 

After oral exposure, the gastrointestinal tract absorbs approximately ninety-five percent of ingested MeHg. MeHg can be also absorbed by inhalation and about 80% is retained after vapor exposure. In the bloodstream, it can establish specific chemical bonds with tissue proteins, coupling covalently to selective groups by means of large neutral amino acid transporter. Indeed, MeHg is bound to active sulfhydryl (–SH) groups of e.g., cysteine or glutathione in a 1:1 ratio, forming in the first case MeHg cysteinate (MeHg-Cys) complexes of defined stoichiometry, responsible for the toxicological behavior of this compound. Therefore, the distribution from blood to tissue is slow and the equilibrium occurs approximately four days after exposure [18].

Approximately 10% of the body burden is in the head region; despite the fact that its uptake into the brain is slower than for other organs, the brain system has stronger affinity for MeHg, showing concentrations 3–6 times higher than in the blood. About 20% of the MeHg present in the brain is water-soluble and mainly found as MeHg-glutathione complex [18]. 

Via enterohepatic circulation, MeHg is subsequently transported out of liver cells into bile that, together with the kidney, represents the main excretion pathway of MeHg [19]. Finally, it is excreted as mercuric Hg in the feces that are the major route for MeHg excretion.

MeHg has a relatively long half-life of approximately 70–80 days in the human body; it is typically measured in blood, cord blood, or hair that reflects only exposure to organic Hg [15]. Concentration of MeHg occurs in the brain, liver, kidneys, placenta, and fetus, especially in the fetal brain, as well as in peripheral nerves and bone marrow [20]. 

Hg exposure represents a significant concern during the course of pregnancy, because of the risk to the fetus. In particular, MeHg readily passes across the placental barrier to the fetus where, binding to hemoglobin, its main ligand in red blood cells, it reaches blood concentrations higher than those of the mother [21]. Within the vulnerable fetal brain, MeHg interacts subtly with the tubulin, the main protein of neuronal microtubules, and triggers a series of alterations in fundamental developing processes, such as cellular division, differentiation, and migration, which may finally lead to cell damage and eventually to cell death [18]. 

The gestational period therefore seems to be a critical “window of exposure” for children [22], with irreversible damage to their nervous system after birth. Symptoms ranging from defective cerebral nerve development to retarded growth have been observed. Such signs and/or effects seem to be visibly absent in mothers. 

## 3. Health Effects and Clinical Presentation

The complexity of the detrimental effects potentially related to a long-term or acute Hg exposure can considerably complicate accurate diagnosis, due to the ability of various Hg forms to deposit in many parts of the human body and alter a wide array of molecular pathways [16]. 

The resulting clinical picture can be differently associated to over 250 symptoms, involving the neurological, renal, respiratory, gastrointestinal, cardiovascular, hepatic, reproductive, and immune systems, with fetotoxicity and genotoxicity in humans [14]. 

While the kidneys are the site of the highest levels of inorganic Hg [17], deposits of metallic Hg are found in the brain, thyroid, breast, myocardium, muscles, liver, kidneys, skin, pancreas, and may be associated with some dysfunction of those organs. The brain and nervous system undoubtedly represent the primary MeHg target tissues for adults, children and neonates [23].

As for occupational exposure, inhalation of Hg vapor is the most important route of contamination, while dental amalgam fillings, which consist of 50% Hg, are a significant source of exposure to Hg^0^ in the general population [3,13]. In this context, neurological/neurobehavioral signs and symptoms, such as depression, paranoia, extreme irritability, hallucinations, inability to concentrate, memory loss, hand tremors, weight loss, perpetually low body temperature, drowsiness, headaches, insomnia, and fatigue, may also occur [17].

Moreover, kidney injuries, including nephrotic syndrome, tubular dysfunction, and glomerular disease have been observed in humans after prolonged exposure to skin whitening creams, laxatives, Hg salts, and fluorescent tubes [3,24]. 

Pervasive chronic low-level Hg exposure may also lead to a range of harmful cardiovascular consequences in humans, such as cardiomyopathy, hypertension, coronary heart disease, myocardial infarction, cardiac arrhythmias, cerebrovascular accident, ischemic heart disease, and generalized atherosclerosis [14,25]. In addition, environmental exposures to Hg are correlated with serum levels of autoantibodies and autoimmunity [26]. 

Finally, since there is no conclusive data, Hg and inorganic compounds are not classifiable according to the International Agency for Research on Cancer (1993) as to their carcinogenicity in humans (Group 3), with the exception of MeHg compounds evaluated as possibly carcinogenic (Group 2B) [27].

The most important correlations between Hg toxicity and various human organ systems are briefly listed in Table 1.

## 4. The Role of the Genetic Background in Mediating Individual Mercury Susceptibility

As mentioned above, the study of environmental genetics, which analyzes the relationship between genes and environment, is providing an additional opportunity to clarify the basis of individual vulnerability to Hg toxicity, despite it is clear that the genetic background is only one of several factors influencing human susceptibility to Hg-related outcomes. In this context, a person’s complete genomic sequence with its molecular differences at the DNA level (genotype) may create individuality (phenotype), acting in combination with environmental influences, while genes and their products do not function independently, but they participate in complex, interconnected pathways, networks and molecular systems that interact between themselves (synergistic effect). 

**Figure 1:**
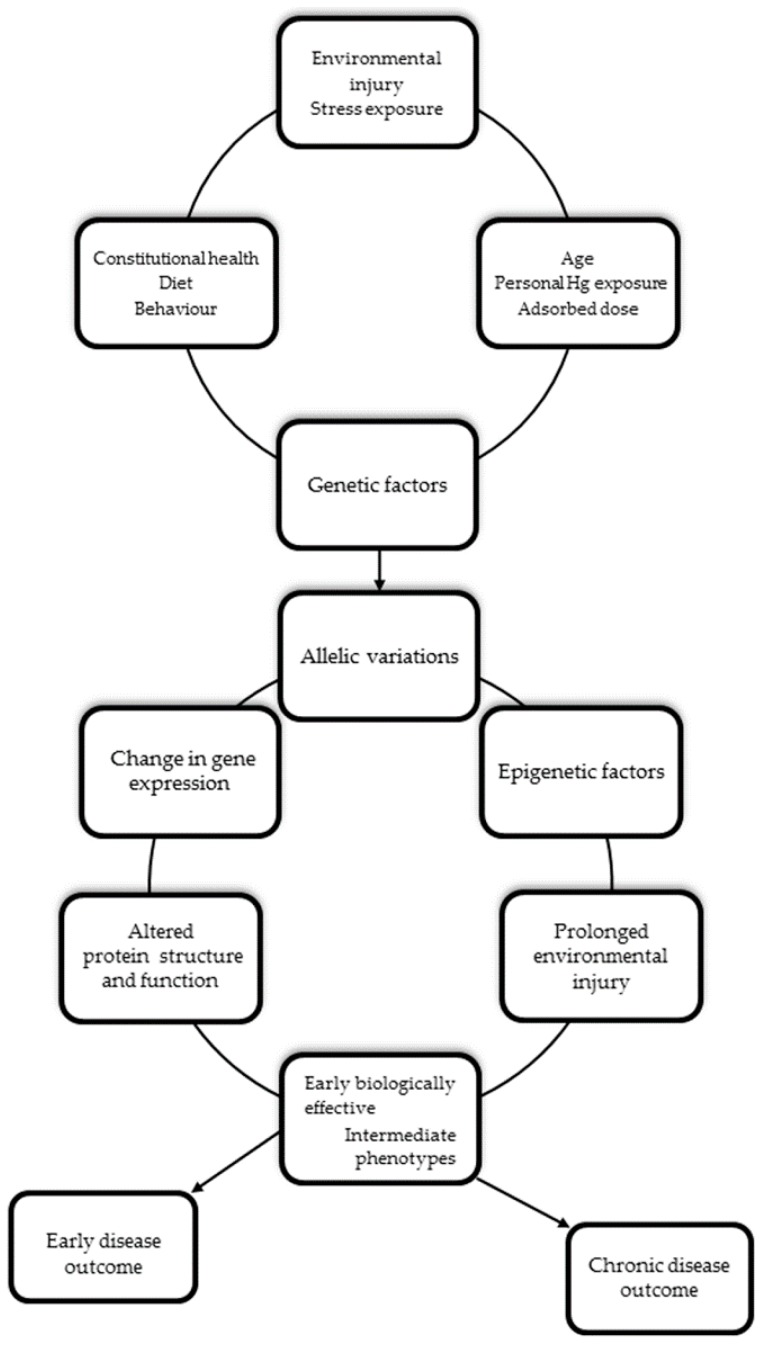
The expanded environmental-genomic heuristic model related to mercury. Genetic and environment interactions contribute to the development of pathological conditions. Allelic variation underlies functional differences in expression or activity of involved gene products and activation of associated biological pathways, leading to manifestation of early or late phenotypic variations. With prolonged exposure to environmental stimuli that also induce long-term epigenetic process on a background of genetic susceptibility, altered gene expression profiles enable the transition of an intermediate state to a chronic pathological state

Figure 1 shows a simplified, theoretical model of how genetic variants, environmental risk factors and different personal stressors may interact to shape the repercussions of Hg on human health.

In particular, the human genome contains millions of DNA sequence variants, arbitrarily referred to as polymorphic variations, if they occur in a population with a frequency of 1% or higher [28]. Among these, there are single nucleotide polymorphisms (SNPs: estimated number >10 million), occurring in single bases of DNA sequences, and representing the most frequent type of DNA variation. Small insertions and deletions (indels) and copy number variations (CNVs; variable number of DNA segments) are also evident.

Each genetic variation, classified in relation to a reference genome fully considered as a wild type, may impact on transcriptional regulation in diverse ways, according to the variant’s size, nature, and location relative to the exonic coding, non-coding or regulatory regions of the gene considered [28]. 

More precisely, there are polymorphisms characterized by whole gene deletions, which clearly eliminate any functional enzyme activity, while others may implicate duplications of the entire gene, resulting in higher levels of activity. In addition, there are the synonymous or silent SNPs, which do not change the amino acid composition of the product, and, thus, are unlikely to modify protein activity.

However, emphasis is placed on a particularly important type of variants, the non-synonymous coding SNPs (nsSNPs), occurring in a gene-coding region. Since these latest variants may induce differences in primary molecular structures at the DNA level, they alter the encoded amino acid sequence, thus potentially affecting proteins composition, function, and their interactions with other molecules [29]. 

Many gene-environment studies focused on those nsSNPs playing a significant role in Hg toxicokinetics, in order to clarify, in the first place, the interactions between the genetic makeup and mechanisms of Hg action. In the second place, because these polymorphisms may provide an opportunity to better understand inter-individual susceptibility to Hg outcomes, especially for more susceptible populations. Perhaps, the time is approaching in which we will be able to elucidate the complex interaction between Hg toxicity and the factors modifying its effects.

## 5. Search Strategy and Study Selection

Since this review intends to briefly summarize key results on the relationship between gene variants, Hg toxicokinetics and health outcomes in susceptibly populations (children and adults), we reviewed original epidemiological studies using PubMed and PubMed Health, resources of National Center for Biotechnology Information (NCBI), and U.S. National Library of Medicine 2016 (http://www.ncbi.nlm.nih. gov).

The literature search strategy was based on key words, or combinations of them, related to “mercury” and “polymorphisms”, such as “gene variants and mercury” or “genetic susceptibility to mercury”, “mercury toxicokinetics and genetics”, “human health outcomes and mercury”. 

Initially, peer-reviewed literature were examined, and the references in the studies found have been analyzed to find additional articles. A further review of the abstracts, titles, and keywords led to the elimination of those quotations that did not address the topic.

Then, the most appropriate genes and proteins playing a significant role in Hg toxicokinetics in humans, in addition to biological-molecular data, were selected.

Subsequently a database search was supplemented in the NCBI Gene database (NCBI gene, www.ncbi.nlm.nih.gov/gene), to analyze the human associated Hg genes, and NCBI Short Genetic Variations database (NCBI dbSNP, www.ncbi.nlm.nih.gov/projects/SNP), to identify all SNPs using an “rs” format. 

Each set of studies pertaining to a type of outcome, for example, biomarkers variation of MeHg (hair, blood, and erythrocytes) or inorganic Hg (urine, whole blood, and plasma) exposure, neurological-neurobehavioral effects in children/adults, or cardiovascular damage, was then evaluated, focusing on the impact of functional polymorphisms on related health Hg outcomes in different population groups. As a result, a synopsis of emergent main potential modifier genes, specific SNPs of relevance and, where observable, adverse health effects, is presented in the following subsections. 

## 6. Gene Classes of Interest and Related Polymorphisms

### 6.1. Inter-Individual Variability and Mercury Body Burden

#### 6.1.1. The Glutathione System

Reduced glutathione (GSH-γ-glutamyl-cysteinyl-glycine), an essential tripeptide present in large quantities in all mammal cells, is the main agent of the glutathione detoxification system (GSHs). This system, which neutralizes the free radicals producing reactive oxygen species (ROS), protects cells against damage resulting from exposure to many external agents and oxidative stress. GSH, therefore, fosters a positive body’s response to the negative Hg actions towards natural detoxification process, and it is essential to heal the damage within the cells. 

Various epidemiological studies have suggested that the response to Hg may be influenced by molecular variants in several regulatory GSHs genes, involved in absorption, distribution, metabolism and excretion process, better known as the Hg toxicokinetics, as reflected in Table 2. Consequently, the genetic component of human Hg susceptibility has now become another aspect not to be underestimated in this context [30,31,32,33,34,35,36]. In doing so, subjects with certain GSHs variants may tolerate higher Hg exposures, due to faster elimination and/or better antioxidative, glutathione-associated capacity [37]. Certainly, some polymorphisms of codifying genes for the Glutathione S-transferase family (*GSTs*) play a prominent role in this sense.

*GSTs* (subclasses alpha, mu, pi, omega, theta, and zeta) is a set of cytosolic enzymes with different tissue distributions that catalyze the conjugation of GSH to a wide variety of electrophilic substrates [43]. Several types of allelic variations have been identified within *GSTs* genes cluster, with *GSTM1* (GST-mu1), *GSTT1* (GST-theta1) and *GSTP1* (GST-p1) receiving the greatest attention in genetic epidemiological studies.

The first two genes encode their respective isoforms with impaired catalytic activity, because polymorphically deleted. Consequently, individuals carrying the double–deleted homozygous genotypes for both *GSTT1* and *GSTM1* genes (“null” genotypes: −/−) may present higher levels of intermediates of oxidative metabolism, which unbalance the antioxidant status, finally leading to exacerbation of the pathological effects of ROS [44]. Overall, the ability of the “null” genotypes to express less or no functional enzymes would entail lowered levels of GSH-conjugates [12]. On that basis, the resulting variable responses towards Hg would be reasonably reflected in a reduced excretion and/or increased retention of Hg in hair, blood, erythrocytes and urine among exposed individuals [11]. Critically, these mutations also seem to influence Hg retention in maternal or cord blood, increasing the risk of low birth weight for offspring of those mothers with higher blood Hg level and “null” genotypes [38].

As regards third genes, *GSTP1* encodes a GSHs subunit able to alter the sensitivity to Hg inhibition, by affecting MeHg retention [45]. *GSTP1* is an enzyme of particular interest, as it is the most widely expressed *GSTs* (found in erythrocytes, placenta, lung, brain, muscle, liver, and more). So far, two very important *GSTP1* polymorphisms (rs1138272 and rs1695) in Hg toxicokinetics have been associated to MeHg biomarker levels in epidemiological studies, with multiethnic, multicultural and bio-diverse features [6,10,30,31,32,34,36]. 

Furthermore, glutamate-cysteine ligase (GCL), a first rate–limiting enzyme in the GSH synthesis for the formation of the mature tripeptide, represents a critical determinant to Hg metabolism [12]. The active protein consists of two catalytic and modifier subunits, respectively coded by *GCLC* and *GCLM* genes, with some functionally significant polymorphisms.

Some of these, in synergy, may affect both gene expression and protein levels, depending on the type of allele variant and dose. Minor alleles of two SNPs (rs17883901 in *GCLC*; rs41303970 in *GCLM*), known to decrease promoter activity, have been related to increased MeHg biomarker levels in individuals from Sweden and Austria [30,32,34]. Moreover, the latter polymorphism has been associated to higher whole blood, plasma, and urine Hg levels among exposed Ecuadorian gold miners and merchants, reflective of their occupational exposure to elemental Hg vapor [7,31]. Recently, a British cohort–study has identified the minor allele of another *GCLC* SNP (rs1555903) significantly related to Hg retention in the umbilical cord [39]. 

Instead, there are no conclusive data to confirm two other variants in *GSHs* genes, respectively *GSTA1* (rs3957356) [34], and *GSS* (Glutathione synthetase, rs3761144) [6], located upstream of their respective coding region, may impact on the body’s ability to eliminate MeHg as a GSH–conjugate. 

Interestingly, significant interactions between *GSHs* mutations and common Hg biomarkers for both organic and inorganic Hg fractions often drive towards a final Hg-related direction arising from a complex interplay of synergistic effects, involving particular alleles of different *GSHs* loci. This is the case of two Austrian studies on medical students, where Gundacker and colleagues [33,34] identified some cooperative *GSHs* networks, able to influence the relationships between Hg inorganic/organic exposure and related biomarkers levels, in function of their polymorphisms. Indeed, the co-existence of *GSTT1*−/− and *GSTM1*−/− genotypes appeared associated with higher hair Hg levels than homozygous wild types, whereas the synergistic effect of the dual *GSTP1* rs1138272/*GSTT1*−/− and *GSTP1* rs1695/*GCLC* rs1788390 mutated combinations confirmed and strengthened this association, when compared to single-gene variants. 

Also Goodrich et al. [6] examined inter-individual differences in Hg body burden, always in function of some key *GSHs* polymorphisms, as part of an association study conducted in Michigan dental professionals. For their part, the authors established genetic influences by both *GSTT1*−/− deletion on urine Hg levels, and *GSTP1* rs1138272 and rs1695 polymorphisms on hair Hg levels for this category of workers, in a context of concomitant exposure to elemental Hg through dental amalgams and MeHg through fish consumption.

Previously, a Swedish study had detected this additive effect in carrier subjects of both minor *GSTP1* rs1695 and *GSTP1* rs1138272 allelic variants, however related with a reduction of Hg content in erythrocytes, after controls for polyunsaturated fatty acids levels [32]. 

In addition, de Oliveira et al. [10] tried to elucidate the genetic impact on Hg body burner of highly exposed riverside communities of the Brazilian Amazon, by evaluating their exposure to different Hg species (MeHg and inorganic Hg). The authors suggested a direct involvement of some *GSHs* polymorphisms (*GSTM1* deletion, *GSTP1* rs1695, *GCLM* rs41307970, and *GCLC* rs17883901) on the modulation of MeHg levels in plasma and whole blood.

Meanwhile, Barcelos et al. [9] has previously confirmed the potential synergistic effects of aforementioned *GSTM1* and *GCLM* mutations on MeHg metabolism in the same population, with high exposure from fish intake.

Finally, a very recent cross–sectional study of Parajuli et al. [36] evaluated the interactive mechanism between representative biomarkers for MeHg (hair, blood) and elemental Hg (urine) exposures, and multiple gene pathways involving, for instance, selenoproteins, metallothioneins, and xenobiotic transporters (discussed below) in addition to those concerning glutathione metabolism. 

Their major findings consolidated the interactive effects between six gene variants (*GCLC* rs138528239, *MT1M* rs2270836, *MT4* rs11643815, *ATP7B* rs732774-rs1061472, and *BDNF* rs6265), and emblematic biomarkers levels in a population of dental professionals from the state of Michigan (USA), doubly exposed to MeHg (via fish consumption), and elemental Hg (via occupational practices and personal amalgams). The authors, therefore, recommended careful consideration of such gene-environment factors, to improve the Hg risk assessment on vulnerable populations [36].

In summary, active involvement of a plethora of genetic factors, able to intervene in the basic processes for human Hg susceptibility according to their functional priority, with a well-defined hierarchy could be supposed; that is why further investigation is needed.

#### 6.1.2. The Metallothioneins Superfamily

The body’s natural chelating agents, the metallothioneins (MTs), consist in a class of cysteine-rich low-molecular mass proteins (approximately 30% cysteine residues) with multiple cellular functions, including transport, storage and detoxification of metals. MTs bind heavy metals as metal chaperones by means of rich thiol groups, to maintain their homeostatic regulations in a number of different organ systems. These multipurpose proteins may also play a role in preventing, at least partially, Hg accumulation in the liver and blood, and decreasing renal toxicity [46].

There are four major isoforms (MT-I through MT-IV) identified in mammals, encoded by a cluster of genes. Among these, only MT-I (consisting of seven functional genes: *MT-1A*, *B*, *E*, *F*, *G*, *H* and *X*) and MT-II (*MT-2A* gene) are expressed ubiquitously. Conversely, MT-III expression appears to be restricted to the brain, and MT-IV shows a much more restricted tissue expression in stratified squamous epithelia [46]. 

Over the past few years, *MTs* polymorphisms have been emphasized to study individual susceptibility to heavy metals toxicity, even though only some genomic variations have shown significant interactions with human Hg exposure (Table 2) [34,36,40]. 

A first study among Austrian medical students by Gundacker and colleagues [34] identified in *MT4* rs11643815 polymorphism a significant predictor of Hg body burden, related to increased hair Hg contents. 

A professional cohort-study in Michigan [40] further supported the potential *MTs* impact on different exposure-Hg biomarkers, with results not perfectly overlapping to those obtained by Gundacker et al. [34]. The authors established a relevant play-role of *MT1M* (rs2270837) and *MT2A* (rs10636) variants on the relationship between lower urinary Hg level and personal exposure to elemental Hg.

At the same time, *MT1A* (rs8052394) and *MT1M* (rs9936471) polymorphisms were involved in modifying significantly the hair Hg level, in function of the daily MeHg intake from fish consumption [40]. A very recent study, carried out in the same American cohort by Parajuli et al. [36] confirmed a similar trend, with significant main effects in hair and blood Hg levels, however, involving the minor allele of the *MT1M* (rs2270836) and *MT4* (rs11643815) variants.

From a clinical perspective, the molecular effects of these polymorphisms are still not fully understood. However, since all these variants are located in gene positions, such as the coding or 3’untranslated (3’UTR) region, that are very important for regulating transcription and transduction, it is likely that they may alter the molecular structures of different isoforms, affecting Hg retention in terms of metal–binding capabilities, and subsequently biomarker levels [40]. 

Finally, only few association studies on *MTs* polymorphisms have been reported to date concerning somatic traits, such as those of the neuropathological sphere related to Hg exposure (see Table 3 on page 17). In this regard, a relevant epidemiological study carried out among Portuguese children and adolescents [47] showed significant association between two relatively common *MTs* gene variants (*MT1M*, rs2270837; *MT2A*, rs10636) and adverse effects on multiple neurobehavioral functions, principally among boys with chronic elemental Hg exposure. From a cognitive point of view, also a UK birth cohort-study of Julvez e colleagues [39] confirmed a significant functional effect of *MT2A*rs10636 SNP.

#### 6.1.3. The Selenoproteins Family

The concomitant presence of selenium (Se) in several key districts of the human body seems to play an important role in the storage of Hg and its tissue distribution [48]. This essential micronutrient exerts its biological functions predominantly by means of Se-dependent proteins, termed Selenoproteins, involved in a wide range of pleiotropic effects. 

In fact, according to a preferential selenoprotein hierarchy, Se is incorporated into the primary structure at the twenty-first amino acid position as selenocysteine (Secys), analogous to the amino acid cysteine (Cys) in its molecular structure, with an atom of selenium replacing that of sulfur in Cys [49].

Selenoproteins surely play two important roles in protecting against Hg toxicity. First, they may bind more Hg through their highly reactive selenol group, and secondly, their antioxidative properties help eliminate the reactive oxygen species induced by Hg in vivo [50].

Among these synergies, elevated concentrations of MeHg may directly downregulate the expression of many selenoprotein-coding genes, commonly defined as selenogenes, leading to the depletion of potentially important metabolites for the organism’s response to metal toxicity [49].

Is it the case of genes codifying for the glutathione peroxidases class, a family of antioxidant enzymes typically containing one Secys residue, including cytosolic glutathione peroxidase (*GPX1* gene) and phospholipid hydroperoxide glutathione peroxidase (*GPX4* gene). 

Instead, the *SEPP-1* gene codifies for selenoprotein P1, the major protein for selenium transport in the blood. With an amount of up to ten Secys residues carried by the full-length isoform, selenoprotein P1 is thus particularly equipped for binding more Hg through these numerous highly reactive selenol groups [51].

To date, few studies have effectively investigated selenogenes variants and their relationships with Hg biomarker levels (Table 2), despite the fact that both Hg exposure [50] and seleno-SNPs [52] can affect protein expression and activity levels.

From a molecular point of view, two relatively common *SEPP-1* SNPs (rs3877899 and rs7579), located within the protein coding region and 3′UTR of the gene, were emphasized as potential modulators of human selenium metabolism, able to affect both isoform prevalence and gene expression. In this context, the variation in these parameters could, thus, reduce protective binding of selenoprotein-Hg and/or alter Hg distribution to various tissues such as the kidney or brain [52].

Similarly, another *SEPP-1* 3′UTR polymorphism (rs7579) showed a significant major effect on urine and hair Hg levels, following exposures to elemental Hg (via dental amalgams) and MeHg (via fish consumption) in dental professionals from Michigan [6]. In the previously mentioned cohort, Goodrich et al. [53] subsequently observed decreased methylation in a potentially labile promoter region of *SEPP-1* gene, significantly associated with higher hair Hg levels among males, exposed to MeHg.

All these molecular modifications involving selenogene polymorphisms, as well as new effects of epigenetic regulation, may presumably alter the delicate balance between Se and Hg status, with complex and interactive effects on human health that require further genetic investigation in the whole selenoprotein metabolic pathway.

#### 6.1.4. The Xenobiotic Transporter Proteins Superfamilies

Despite the fact that no specific exclusive transporters for Hg have been identified so far, as already mentioned, Hg atoms have a coordination ability to form complexes with proteins, peptides, or amino acids, such as GSH-Hg or Hg-Cys conjugates. As a result, these interactions may give rise to specific substrates for transporter proteins, which are thus involved in the cellular import/export of different forms of Hg across cell membranes [5].

The ATP-binding cassette transporter superfamily (ABCs) provides probably a clear example of these potential transporters related to Hg. This is a system of ATP-dependent pumps, able to move inorganic ions, metals, peptides, steroids, and many other small molecules across cells surface membrane, by means of innumerable components. Among these, some are especially significant also in the clinical context, in terms of transport of endogenous substrates, cancer chemotherapeutics, numerous drugs and metabolites across the plasmatic and intracellular membranes [54].

High levels of some of the best-characterized members, also known as the multidrug resistance-associated proteins (MDR1, MRP1 and MRP2, respectively codified by *ABCB1* gene, Sub-Family *B*; and *ABCC1*–*ABCC2* genes, Sub-Family *C*) are found in BBB, placenta, liver, gut, and kidney. As a whole, the human genome carries 49 *ABCs* genes, arranged in seven subfamilies and designated from *A* to *G*. Functional suppression of *ABCs* activity increases Hg content in cells and sensitivity to Hg toxicity in vitro. 

Furthermore, two other multi-specific transporter families, such as organic anion transporters (OATs), and system L-amino acid transporters (LATs) have been also involved in Hg transport [55]. 

Eleven members of OATs family, encoded by the solute carrier (*SLCs*) genes, are expressed in various tissues with different transport functions for various clinical drugs and endogenous nutrients. In particular, OAT1 (*SLC22A6* gene) and OAT3 (*SLC22A8* gene), expressed on the basolateral membrane of the proximal tubule, are well-studied organic anion transporters. In vivo studies demonstrated their involvement in the cellular uptake of Hg conjugates in multiple districts [55].

Regarding LATs (*SLC7* genes), these proteins transport amino acids into cells in exchange for other amino acids. LAT1 (*SLC7A5*) and LAT2 (*SLC7A8*) can transport the MeHg-L-Cys complex across BBB and placenta, operating through a highly specific mechanism that imitates the L-isomer of certain amino acids, like methionine or homocysteine [55]. 

Some of these active transporters may also influence both inorganic and organic Hg uptake, distribution, and elimination at molecular level, modulating the cellular Hg efflux [5,41,42]. 

Engström et al. [5] provided the first evidence suggesting the possible involvement of potential Hg-transporter gene variants in the inorganic Hg toxicokinetics. This population-study from different countries has positively (*ABCC2*, rs1885301 and rs717620; *SLC7A5*, rs33916661) or negatively (*ABCC2*, rs2273697, *SLC22A6*, rs4149170; *SLC22A8*, rs4149182) associated some transporter genes variants with the urinary Hg excretion among gold miners highly exposed to Hg vapor in Africa (Tanzania and Zimbabwe) and Asia (Indonesia and Philippines) [5]. 

Instead, Llop et al. [42] have strengthened the hypothesis of the involvement of *ABC*s polymorphisms in MeHg body burner modulation, with a study based on two European birth cohorts from three Mediterranean countries (Italy, Greece and Spain) involving both mother and fetus during pregnancy. The authors demonstrated a different relationship between maternal fish intake and cord blood Hg concentrations, depending on the child’s genotype in some *ABC* genes (*ABCB1* rs2032582, *ABCC1* rs11075290 and *ABCC2* rs2273697) [42]. Their results confirmed the existence of personal molecular mediation on MeHg transport across the placenta and accumulation during early development, influencing both Hg internal dose and neurotoxicity. 

Finally, attention must be drawn on the very recent study by Engström et al. [41], based on a mother-child cohort from the Seychelles, a typically population suggestive of high fish intake and hair Hg concentrations. The authors identified in additional *ABCC1* SNPs (rs215088, rs11075290, rs212093), *ABCC2* (rs717620), and *ABCB1* (rs2032582, rs1202169, rs10276499) some probable susceptibility markers to modulate the MeHg transport, distribution and elimination, based on the strong association of their genotypes with maternal hair Hg concentrations. Among them, only the first aforementioned *ABCC1* intronic variant, located in a promoter-flanking region, also showed adverse association with neurodevelopmental outcomes in children [41]. 

These findings are thus promising. However, the real functional correlation between multi-specific transporter gene variants and Hg toxicokinetics (Table 2), directly or indirectly impacting on neurodevelopmental outcomes (Table 3), remains essentially unclear. The results highlighted above indicate that also this topic deserves further investigation.

### 6.2. Genetic Susceptibility and Neurodevelopmental Mercury Outcomes

The concern that prenatal Hg exposure might seriously impair cognitive development in fetuses, infants, children, and adolescents has inspired some talented investigators for many years [17,56,57,58]. Although still debatable, scientific evidence is generating growing awareness that the genetic substratum may play a role in modulating those neurotoxic effects deriving from Hg exposure, mainly related to the organic form.

Unfortunately, it is by no means straightforward to clarify the real links between inter–individual response and Hg-related neurological deficits, moving within susceptibility genes and complex molecular pathways. So far, various theories have been employed to shape these delicate relationships. The presence of some genotypes, able to identify those genetically predisposed children who will be relatively less resistant and consequently more vulnerable to adverse neurobehavioral effects of Hg exposure, could be of course invoked from a genetic perspective.

To this purpose, the case study carried out by Julvez and colleagues [39] is an interesting example. The authors evaluated the presence of cognitive deficits in a subgroup of school age British children with respect to prenatal MeHg exposure, without overlooking the presence of interfering issues on this relationship, involving nutritional, sociodemographic, and genetic cofactors. Four variant forms, located in respective genes with biological plausibility for neurodevelopment or metal neurotoxicity (rs2049046 Brain Derived Neurotrophic Factor-*BDNF*; rs662 Paraoxonase1-*PON1*; rs3811647 Transferrin -*TF*; rs1042838 Progesterone Receptor-*PGR*), were significantly associated with increased cognitive consequences in those children who were carriers of all four main mutated alleles [39]. The genetic potential, therefore, seemed to operate a specific population’s stratification in vulnerable subgroups, culminating in various degrees of susceptibility to MeHg neurotoxicity [39].

At the same time, also some studies by Woods et al. [47,61,66,71] conceived an active involvement of some organized molecular networks, where specific candidate genes may independently favor the onset of detrimental neurobehavioral outcomes in more vulnerable brains of children and adolescents with particular genotypes (see also Table 3). 

In particular, the latest longitudinal evaluation study among Portuguese boys and girls, covering seven years of clinical follow-up, hypothesized that the allelic status of several candidate genes, such as coproporphyrinogen oxidase (*CPOX*), catechol-*O*-methyltransferase (*COMT*), solute carrier family 6 (neurotransmitter transporter, serotonin) member 4 (*SLC6A4*), *BDNF*, *GSTT1*, *MT1M*, *MT2A*, may modulate the Hg effects on their neurobehavioral performance outcomes [61]. To this end, urinary Hg concentrations, as emblematic expression of postnatal inorganic Hg exposure, were related to specific neurobehavioral functions. 

In some cases, the results highlighted statistically significant gene-Hg interactions in specific neurobehavioral domains related to working and learning (the associated variants are detailed in Table 3), whit greater involvement of boys as compared to girls. It is interesting to note that genetic predisposition was clearly able to make a selective distinction of adverse neurobehavioral Hg effects based on gender, possibly triggering more detrimental effects in boys in terms of Hg retention and tissue accumulation [61].

In the current literature there is, therefore, a lot of scientific evidence supporting the role of molecular mediation of neurobehavioral outcomes as a result of chronic low-dose Hg exposure via high consumption of fish, dental amalgams or occupational exposure [62,63,64,67,69,70]. In the light of the recent findings, some genetic variants of plausible candidate genes affecting neurologic functions could alter the orderly functioning of the corresponding neurotransmitters in the human nervous system, in response to the neurotoxic Hg action. 

Mutations in *BDNF* (rs6265), *COMT* (rs4680, rs4633, rs4618, rs6269) and *SLC6A4/5-HTTLPR* (44 bp Ins/Del) genes could show various degrees of involvement during neurodevelopment, based on their biological function, and thus genetically predispose susceptible subjects to different levels of neurocognitive impairment.

Instead, the gene encoding the sixth enzyme in the heme biosynthetic pathway (*CPOX*), which is required for the cellular energy production and formation of neurotransmitters in the brain, may play an indirect role in this context. The most attractive variant between those analyzed (rs1131857 or *COPX4*; rs1729995 or *COPX5*) is a nonsynonymous *CPOX4* mutation in exon 4 of the gene, converting asparagine-to-histidine at amino acid 272 in the protein structure, with the expression of an enzymatic form at lower activity leading to insufficient heme synthesis. Some studies in humans suggested that this variant may increase genetic susceptibility to the adverse neurobehavioral effects of Hg exposure in adults and children [61,66,67], with alteration of urinary porphyrin excretion, a potential biomarker of this effect [65,68]. This would imply a diminished neurotransmitter formation and capacity for neurobehavioral performance [68,72]. 

On the other hand, others polymorphisms could instead predispose some brain regions to reaching increasingly critical levels of Hg, preventing them from performing neurobehavioral functions, particularly in delicate life stages [34,40,47]. 

This is the case, for example, of inherited apolipoprotein-E (Apo-E2, E3 or E4) isoforms, codified by *APOE* gene, recognized as a major risk factor for complex forms of AD, mainly in sporadic late-onset cases [73]. The versatility of this protein arises from having three isoforms changing from one another, because different amino acids are present at position 112 and 158: Apo-E4 has an arginine in position 112 and 158, where Apo-E2 has two cysteines, and Apo-E3, the most common variant, an arginine and a cysteine. Interestingly, cysteine contains a sulfhydryl, which is capable of binding metals, especially bivalent metals like Hg. This means that only the E2 and E3 isoforms, namely those supplied with cysteine rests, could better bind and detoxify MeHg when it crosses BBB and causes damage in the brain.

In contrast, the E4 isoform, which is therefore devoid of sulfhydryl groups, probably fails to connect with MeHg, thus performing a less efficient elimination process. Firstly, *APOE* genotyping (ɛ2-ɛ3-ɛ4 alleles) was considered as potential biomarker of susceptibility to Hg neurotoxicity in chronically exposed AD patients, with presumptive Hg-related neuropsychiatric symptoms, to identify subjects at greater risk and possibly forestall subsequent neurological deterioration [74].

Later on, in a prospective cohort study conducted in Taiwan by Ng et al. [59] the possibility that the presence of *APOE ε4* allele may enhance the risk of behavioural deficit among preschool children, previously characterized by elevated Hg concentrations in the cord blood, was investigated. The findings confirmed the influence of *APOE* on the child’s neurodevelopment by means of three allelic variants ε2, ε3 and ε4 (rs7412 and rs429358), acting as important protective (ε2) or risk (ε4) factors [60].

Given the prognostic potential of these studies on the gene-environment interactions, it is hoped that any resulting interference by Hg exposure on the critical human brain functions may be fully unveiled, in order to protect the most susceptible individuals, presenting increased sensitivity to neurobehavioral deficits. 

Table 3 shows the abovementioned genes and associated molecular markers to deficits on performance within the neurobehavioral domains, symptoms, and mood in different study populations. 

### 6.3. Genetic Susceptibility and Cardiovascular Mercury Outcomes

Although the cardiovascular system is not typically a primary target of Hg, currently there is increasing concern regarding the impact that Hg could have on this system, even at low doses of exposure. In fact, Hg is considered an important risk factor for cardiovascular disease, playing a role in the development of cardiovascular events [75,76,77]. So far, the mechanisms underlying these Hg effects have not been entirely clarified, however, the most accredited hypothesis supports an increased oxidative stress due to excessive production of free radicals.

Suggestive epidemiological studies in Finland [78] firstly demonstrated that people with high Hg levels in biomarkers of exposure may be identified as “at risk” of progression for several cardiovascular disorders [79]. Therefore, it would be of great significance for public health to include this endpoint in the regulatory for global Hg risk assessment [80]. 

Instead, clinical data supporting the hypothesis that genetic polymorphisms can modulate human susceptibility to Hg–induced cardiovascular damages are still incipient and not consistently confirmed (for more details see Table 4).

Jacob-Ferreira et al., who previously associated plasma Hg levels with increased circulating net Matrix metalloproteinase (MMP)-2 and MMP-9 activities [81], demonstrated the involvement of some *MMPs* genetic variants, in modulating proteins expression or activity towards important pathological cardiovascular consequences in exposed subjects, based on Hg intake. The authors identified significant correlation between plasmatic Hg levels and two functional *MMPs* polymorphisms, a *MMP-9* promoter (CA)_n_ repeats (rs 3222264) and a *MMP-2* promoter polymorphism (rs243865), which likely increased the cardiovascular risk by affecting circulating MMPs levels in fish-consuming Brazilians [82,83]. 

Some variants in the endothelial nitric oxide synthase 3 (*NOS3*) gene, codifying an antithrombotic and anti-atherogenic protein, which is the main source of a powerful vasodilator in the vascular wall (nitric oxide, NO) were subsequently involved in the same context of riverside communities of the Brazilian Amazon. The authors identified the contribution of the 27-pb variable number tandem repeat (VNTR) polymorphism in the *NOS3* intron 4 (27 bp VNTR-4a/4b) in enhancing susceptibility to cardiovascular disease after MeHg exposure, hypothesizing a strong impact on plasma and blood nitrile levels [84,85].

Conversely, two other *NOS3* polymorphisms, respectively located in the promoter region (rs2070744) and in exon 7 (rs1799983) of the gene, were not affecting the NO bioavailability in the same Hg-exposed Brazilian population [86]. 

Finally, a Swedish case–control study [37] suggested the potential impact of the relatively rare *GCLM* genotype (rs41303970) on the association between both Hg levels in erythrocytes/protective fish agents (n-3 polyunsaturated long chain fatty acids) levels in plasma, and risk of first ever myocardial infarction, advocating larger epidemiological investigations to confirm this joint hypothesis.

## 7. Discussion

With the advances in genetics and high-quality information on Hg pollution, current research has shown that the interplay between genes and environment is critical to better understand the Hg impact on human health [87]. Despite the great potential, there has, however, been little use of genetic information in Hg risk assessments for both occupational and environmental exposures.

So far, the base scientific evidence involving genetic interference in modes of Hg action and adverse outcomes has been limited to predisposing Hg risk factors located in genes already known for interactions in Hg toxicokinetics. In this perspective, polymorphic gene variants in the GSHs metabolic pathway have received considerable attention, due to their ability to influence Hg metabolism, by selectively altering the corresponding enzymatic activities or gene expression.

In particular, some *GSTs* polymorphisms might differently impact on the elimination of methyl-Hg and inorganic Hg, and thereby affect the Hg body burden, just like the dual gene combination of allelic *GCL* and *GST* variants might intervene in this sense, by acting in synergy on Hg biomarker levels. Similarly, molecular variants of the MTs, selenoproteins, ABCs, LATs and OATs biological pathways might interact with environmental exposures to jointly influence Hg disease.

However, several not always perfectly overlapping interpretations of results were provided by well-designed studies, based on significantly exposed subjects to both MeHg and inorganic Hg, often even with the same ethnic background. In this respect, it is worth pointing out that the genetic polymorphisms differ from other established prognostic markers generally used in studies of this type, because they have the unique feature to be unchangeable in lifetime. Consequently, the detection of genotypes associated as markers of Hg susceptibility is an inalterable phase that cannot be modified during Hg exposure.

On the contrary, a range of other variable factors may be critical, and interfere on data collection and analysis, partially justifying the inconsistent results. For example, the selection bias is an important problem to evaluate. The homogeneity of genetic background or, vice versa, the heterogeneous substructure of the cohort investigated with different allele frequencies of subpopulations, due to an ancestry difference, the appropriate statistical methodology and analysis, the correct power to detect an association are, undoubtedly, all crucial factors to validate genetic evidence.

Another aspect that should not be underestimated is a targeted selection of candidate-genes, which remains subjective although based on criteria of biological plausibility, and identification of related genetic variants, as well as possible impact of linkage disequilibrium and/or potential presence of epistasis should be properly evaluated.

In addition, the use of misclassified or biased information on Hg exposures might be an additional problem, concerning such epidemiological studies. A retrospective evaluation of Hg impact on human health should indeed take into account its dynamic interactions with all those other variables, arising not only from the essence of Hg itself (i.e., source, duration and levels of exposure, speciation, corresponding biomarkers analyzed, dietary intake, work activities), but also from environmental disturbances. For example, simultaneous exposure to multiple heavy metals could trigger additive or synergistic effects on the final health outcomes, with marked influences in the estimate of individual risk not entirely attributable to Hg exposure.

Such concepts may also be shifted to the neurological sphere. The relationship between the gene-Hg interactions, neurobehavioral development, and neurotoxicity is the most obvious example, especially in infants and children, where individual differences are most salient since the extent of impairment is necessarily individual. 

Actually, how the Hg interacts with genetic factors to alter the human susceptibility remain poorly understood also in this context. However, significant attention has been directed toward some genetic variants in candidate genes, such as *CPOX*, *COMT*, *BDNF*, *SLC6A4*, *PGR*, *APOE*, given their well-known involvement in different cognitive mechanisms and neurodevelopmental disorders, with particular emphasis to neurotoxicity caused by MeHg. In some instances, these variants may intervene strategically in the detrimental Hg mechanisms from the earliest stages of life, by influencing the personal response to neurotoxicity also with latent effects and, thus, modulating neurobehavioral and cognitive performances through childhood and adolescence.

With its background of crucial mediating factor in neurological and cognitive processes, the *APOE* ε4 allele is a major genetic risk factor investigated for its association not only with dementia forms, but also with normative cognitive development. So far, major studies have been conducted on adults, while fewer studies explored this aspect in children and young people, especially if Hg-exposed. However, Ng et al. [59,60] suggested that *APOE* ε2/ε4 alleles could play a different role in modifying the effects of cord blood Hg in children prenatally exposed on their neurodevelopment. At the same time, the hypothesis tested by Woods et al. [61] upon the existence of interactions between postnatal Hg exposure and *APOE* ε4 has confirmed significant genetic modification of Hg effects on neurobehavioral domains, related to working and learning memory in young people.

Meanwhile, increased Hg accumulation had been identified in brain tissue for *APOE* ε4 carriers, speculating that the well-known differential genetic epidemiology of *APOE* might play a part in the differential capacity of Hg detoxification [74], entailing a greater risk of developing Hg-induced neurological outcomes in these subjects across the lifespan. 

In addition to the *APOE* ε2/ε4 polymorphisms in coding sequence, *APOE* contains, however, other interesting functional polymorphisms in the promoter at multiple regulatory sites, able to influence gene transcription, and finally the quantitative expression of APOE levels. More specifically, promoter is a section of a special DNA sequence, which is located at the upstream of a gene and participates in the regulation of the biological activity of the responding gene. 

Therefore, we suggest that the consideration of haplotype-based analysis involving structural polymorphisms in the *APOE* promoter area, in addition to the functional variants located in the gene-coding region, may be helpful in terms of a more accurate individual risk assessment. 

In this framework, additional data and evidence should also involve new functional polymorphisms in interactively-candidate genes, for example through genome-wide association studies, to clarify the genetic basis of personal vulnerability to Hg toxicity, and assess the clinical relevance particularly on prenatally exposed subjects.

Nowadays, rapid technological advances involving SNPs genotyping, next-generation sequencing or gene expression profiling may offer precious information on the predictive potential of millions of key variants in overlapping biological networks.

Looking forward, better comprehension of genetic variations in response to detrimental Hg action would have positive implications in the near future, starting with:

- Understanding of the underlying biological Hg mechanisms;
- Clarification of the variability and critical windows of susceptibility in the development of Hg-related health outcomes;
- Implementation potential preventive measures, and treatment;
- Improved Hg risk assessments and decision-making;
- Reduced global health disparities;
- Enhanced quality of life of all people.

For instance, an evaluation of the functional link between genotype and phenotypic differences may allow defining real individual responses to Hg effects, by narrowing the gap between perceived and true health risks from the world’s population. 

Secondly, this information may be exquisitely effective in alleviating human health concerns linked to potentially hazardous situations for minority groups “at high risk” of Hg exposure, such as fishing communities living in coastal areas, or children employed in the artisanal and small-scale gold mining in rural areas.

Finally, the possibility of characterizing “genetic profiles” of susceptibility in asymptomatic individuals may represent a further positive aspect, particularly relevant for all cases of chronic Hg poisoning, who may be at risk of progression for neurological and cardiovascular impairments. In this context, longitudinal birth cohort studies enrolling pregnant women with repeated measurements of different exposure-Hg biomarkers, collection of biological samples since birth over time, and comprehensive clinical/neurological Hg outcome assessments could have great potential, complemented by targeted genetic screening.

Of course, the application of new genetic information to Hg risk assessment and disease prevention efforts will be technically difficult but, perhaps, is even more challenging from an ethical, legal and social point of view. However, great progress can be expected in the next decade.

## 8. Conclusions

According to the epidemiological studies carried out so far, a personal vulnerability to detrimental Hg action due to individual genetic makeup appears to be an interesting hypothesis to strengthen and interpret different repercussions on human health arising from the release of Hg in the environment.

In this context, the elucidation of molecular mechanisms involved in imparting toxicity by Hg may be very essential. The hope is that new information will be used to implement a comprehensive public health action plan in a context of global Hg risk assessment, for identifying and protecting susceptible individuals from Hg hazards.

Although the goal is challenging, more focused investigations characterized by improved Hg exposure analysis, rigorous epidemiologic study design, advanced genomic technology and a better understanding of Hg toxicokinetics will help to bridge this knowledge gap. While genetic susceptibility is clearly important and more studies are needed to identify increasingly specific interactions, high priority should, however, begiven to reducing Hg pollution exposure in general. 

## Figures and Tables

**Table 1:** Systemic toxicological effects and symptoms of mercury poisoning.

Human Organ System	Exposure Pathway	Marker	Associated Outcome
Nervous system	Transplacental for maternal occupational settings or dietary intake. Breast-feeding and ingestion of contaminated food. Inhalation, for occupational exposure. Use of thimerosal in vaccines. Cultural or religious practices. Dermal exposure. Chronic exposure from amalgam dental fillings. Inhalation, for occupational exposure or dental amalgams replacement. Ingestion of contaminated food, particularly in fish eating populations.	Cord blood. Maternal milk, hair and blood. Child blood, hair and urine. Adult urine, blood and hair.	Infants/Children: Deficit in language (late talking) and memory, deficit in attention, decrements in infant cognition and neurobehavioral deficits. Adults: Depression, paranoia, extreme irritability, hallucinations, inability to concentrate, memory loss, tremors of the hands, head, lips, tongue and eyelids, low body temperature, drowsiness, headaches, weight loss, insomnia, fatigue, blindness, optic neuropathy, retinopathy, hearing loss, sensory, neurological and behavioral dysfunctions.
Motor system	Pre and post-natal exposure. Ingestion, for fish eating populations. Inhalation, for gold mining activities.	Maternal hair. Adult blood, hair and urine	Motor dysfunctions, increased tiredness, reduction in muscle strength and twitching, late walking.
Cardiovascular system	Chronic exposure, attributed to fish consumption and gold mining activities.	Adult hair, toenail, plasma and urine	Cardiomyopathy, hypertension, coronary heart disease, myocardial infarction, cardiac arrhythmias, cerebro-vascular accident, ischemic heart disease, generalized atherosclerosis.
Pulmonary system	Inhalation, for chronic exposure of volatilized vapors. Inhalation, for burning of Hg-containing material.	Adult urine	Chemical pneumonitis, necrotizing bronchitis, pulmonary fibrosis, cough, dyspnea, chest tightness, asthmatic disorders.
Renal system	Occupational contact, for abuse of skin lightening cosmetics or Hg-containing compounds. Chronic exposure, related to the number of dental amalgam fillings. Chronic dietary exposure, for fish consumption.	Adult urine, hair and blood.	Glomerular disease whit oliguria or anuria, increased plasma creatinine level and proteinuria. Subacute-onset nephrotic syndrome, nephritic syndrome, tubular dysfunction, glomerulonephritis.
Endocrine/Reproductive system	Prenatal exposure for maternal amalgam fillings/replacement. Chronic exposure from amalgam dental fillings. Exposure to occupational routes	Child urine and blood. Adult urine, hair and blood.	Adrenal hyperplasia and atrophy. Hypothyroidism, thyroid inflammation, and depression. Pancreatic dysfunction. Decreases rate of fertility in both males and females. Birth of abnormal offsprings.
Immune/Hematological system	Chronic dietary exposure. Clinical hypersensitivity for Hg-containing amalgam. Prolonged exposure in clinically asymptomatic workers.	Adult urine. Lympho/monocyte stimulation tests.	Induction and exacerbation of autoimmune and allergic diseases in susceptible populations. Decreased immunity of the body. Hemolytic anemia, aplastic anemia.
Embrional system	Maternal occupational settings. Maternal dietary exposure. Maternal amalgam fillings/replacement.	Cord blood. Fetal blood. Maternal hair.	Hypoplasia of the cerebellum, decreased number of nerve cells in the cerebral cortex, decreased total brain weight, abnormal neuron migration. Spontaneous abortions, stillbirth, low birth weights.

**Table 2:** Relevant genes and their potential role for mercury toxicokinetics, with the most recognized markers of susceptibility to mercury exposure.

Gene	Protein Function	Polymorphism	“In vitro” Functional Activity	Estimated Interactions	Mercury Metabolism
***GSTM1***	Hg-binding modulation by GSH conjugation	+ > – Deletion	Null gene expression and impaired catalytic activity	Urinary Hg levels	I-Hg ^1^ [31]
				Hair+ Blood Hg levels	Me-Hg ^2^ [9]
				Hair Hg levels	Me-Hg [33,34]
				Maternal hair Hg levels	Me-Hg [38]
				Cord blood Hg levels	Me-Hg [38]
				Plasma/Blood Hg levels	Me-Hg [10]
***GSTT1***	Hg-binding modulation by GSH conjugation	+ > – Deletion	Null gene expression and impaired catalytic activity	Urinary Hg levels	I-Hg [6,7]
				Hair Hg levels	Me-Hg [33,34]
				Cord blood Hg levels	Me-Hg [38]
***GSTP1***	Hg-binding modulation by GSH conjugation	105 Ile > Val (rs1695)	Decreased catalytic efficiency and protein activity	Urinary Hg levels	I-Hg [31,32,33,34,35,36]
				Hair Hg levels	Me-Hg [6,7,8,9,10,11,12,13,14,15,16,17,18,19,20,21,22,23,24,25,26,27,28,29,30,31,32,33,34]
		114Ala > Val (rs1138272)		Erythrocyte Hg levels	Me-Hg [30,31,32]
				Plasma Hg levels	Me-Hg [10]
***GSTA1***	Hg-binding modulation by GSH conjugation	3′UTR (rs3957356)	Lower transcriptional activation and decreased enzyme activity	Blood Hg levels?	Me-Hg [34]
***GSS***	Hg-binding modulation by GSH synthesis	5’UTR (rs3761144)	Decreased gene expression	Hair Hg levels	Me-Hg [6]
***GCLC***	Hg-binding modulation by GSH synthesis	5′ near gene (rs17883901)	Suppressed gene induction and promoter activity	Urinary Hg levels	I-Hg [31]
				Hair Hg levels	Me-Hg [33,34]
		Intron 9 (rs1555903) *		Erythrocyte Hg levels	Me-Hg [30]
				Blood Hg levels	Me-Hg [10,11,12,13,14,15,16,17,18,19,20,21,22,23,24,25,26,27,28,29,30,31,32,33,34,35,36]
				Cord blood Hg levels *	Me-Hg [39]
***GCLM***	Hg-binding modulation by GSH synthesis	5’ near gene (rs41303970)	Decrease promoter activity and gene expression	Urinary Hg levels	I-Hg [7,8,9,10,11,12,13,14,15,16,17,18,19,20,21,22,23,24,25,26,27,28,29,30,31]
				Hair, Blood Hg levels	Me-Hg [9,30,37]
				Plasma, Blood Hg levels	Me-Hg [10]
				Erythrocyte Hg levels	Me-Hg [32]
				Plasma Hg levels	I-Hg [7,8,9,10,11,12,13,14,15,16,17,18,19,20,21,22,23,24,25,26,27,28,29,30,31]
***MT1M***	Hg-binding and detoxifying capacity	3′UTR (rs2270837) *	Altered transcriptional activity	Urinary Hg levels *	I-Hg [40]
		3′UTR (rs2270836) *		Hair, Blood Hg levels *	Me-Hg [36]
		3′UTR (rs9936471) *		Hair Hg levels *	Me-Hg [40]
***MT2A***	Hg-binding and detoxifying capacity	3′UTR (rs10636)	Altered transcriptional activity	Urinary Hg levels	I-Hg [40]
***MT1A***	Hg-binding and detoxifying capacity	51 Lys > Arg (rs8052394)	Altered transcriptional activity and protein structure	Hair Hg levels	Me-Hg [40]
		27 Thr > Asn (rs11640851)			
***MT4***	Hg-binding and detoxifying capacity	48 Gly > Asp (rs11643815)	Altered transcriptional activity and protein structure	Hair Hg levels	Me-Hg [34]
				Hair, Blood Hg levels	Me-Hg [36]
***GPX1*** ***GPX4***	Hg-detoxification and modulation by GSH metabolism	200 Pro > Leu (rs1050450)	Defective gene expression and reduced protein activity	Undefined	None
				Undefined	None
		3′UTR (rs713041)			
***SEPP1***	Hg-detoxification and distribution by Se-P synthesis	3′UTR (rs7579)	Impaired gene expression and protein synthesis	Urinary Hg levels	I-Hg [6]
				Hair Hg levels	Me-Hg [6]
***ABCC2 (MRP2)***	Hg-transport and elimination	5′UTR (rs1885301) * (rs717620) *	Defective promoter sites activity and altered protein	Urinary Hg levels *	I-Hg [5]
				Maternal hair Hg levels *	Me-Hg [41]
				Cord blood Hglevels *	Me-Hg [42]
		417 Val > Ile (rs2273697) *			
***ABCB1 (MDR1)***	Hg-transport and elimination	893 Ala > Ser (rs2032582) *	Impaired protein structure and enzyme activity	Cord blood Hg levels	Me-Hg [42]
				Maternal hair Hg levels *	Me-Hg [41]
		3′UTR (rs12076499) * (rs1202169) *			
***ABCC1 (MRP1)***	Hg-transport and elimination	Intron 1 (rs11075290) *	Defective transcription factor binding sites	Cord blood Hg levels *	Me-Hg [42]
				Maternal hair Hg levels *	Me-Hg [41]
		5′UTR (rs212093) * (rs215088) *			
***SLC7A5 (LAT1)***	Hg-uptake and distribution	5′UTR (rs33916661)	Altered transcriptional activity	Urinary Hg levels	I-Hg [5]
***SLC22A6 (OAT1)***	Hg-uptake and distribution	5′UTR (rs4149170)	Altered transcriptional activity	Urinary Hg levels	I-Hg [5]
***SLC22A8 (OAT3)***	Hg-uptake and distribution	5′UTR (rs4149182)	Altered transcriptional activity	Urinary Hg levels	I-Hg [5]
***ATP7B***	Hg-transport?	832 Lys > Arg (rs1061472)	Altered mechanism of copper transport	Hair Hg levels	Me-Hg [36]
		952 Arg > Lys (rs732774)			
***BDNF***	Hg-neurotoxicity?	66 Val > Met (rs6265)	Altered survival of striatal neurons in the brain	Hair Hg levels	Me-Hg [36]

^1^ Inorganic mercury; ^2^ Methyl-mercury; (rs): rs number; *: direct correspondence between SNPs and mercury levels in different biomarkers.

**Table 3:** Summary of main genetic variants related to mercury-induced neurotoxicity by association studies, with potential involvement for human race.

Gene	SNP	Age Class/Place of Origin	Main Outcomes	Estimated Interactions	Exposure
***APOE***	* rs7412	Children (2 years of age)/Taiwan	Adverse effects on cognition, behavior and whole neuro- development in pre-school children carrying the ε4 allele	Cord blood Hg levels	Prenatal MeHg exposure [59,60]
	rs429358	Children (8–12 years of age)/Portugal	Impaired neurobehavioral functions, related to working and learning memory, among boys with ε4 allele	Urinary Hg levels	Postnatal inorganic Hg exposure [61]
***BDNF***	rs6265 rs2049046	Children (8–12 years of age)/Portugal Adult (Dentists and dental assistants)/USA Children (8 years of age)/UK	Increased risk of neuro-behavioral deficits associated with learning &memory	Urinary Hg levels	Postnatal inorganic Hg exposure [61]
			Altered cognitive flexibility, working & visual memory. Potential decline of cognitive & motor performance with increased neuro-behavioral symptoms, and mood	Urinary Hg levels	Occupational elemental Hg exposure [62,63]
			Decreased performance IQ, and verbal scores with cognitive involvement in children at school age	Umbilical cord Hg levels	Prenatal MeHg exposure [39]
***COMT***	rs4680 rs4633 rs4618 rs6269	Children (8–12 years of age)/Portugal Adult (Dental assistants)/USA	Impaired neurobehavioral test performance affecting attentional control, working and learning memory, visual spatial acuity (boys); attention control, learning memory, and executive functions (girls)	Urinary Hg levels	Postnatal inorganic Hg exposure [61]
			Some mood states (tension, depression, fatigue, and confusion) among female assistants	Urinary Hg levels	Postnatal inorganic Hg exposure [64,65]
***CPOX***	rs1131857 (CPOX4)	Children (8–12 years of age)/Portugal	Altered performance on multiple neurobehavioral tests within neurological domains (attention, learning &memory, executive function, visual spatial acuity and motor function) (boys); impaired performance affecting learning & memory, executive function (girls)	Urinary Hg levels	Postnatal inorganic Hg exposure [61,66]
	rs1729995 (CPOX5)	Adult (Dentists and dental assistants)/USA	Deficits in neuropsychological performance within neuro-behavioral (male dentists) and visuomotor (female dental assistants) domains, symptoms and mood	Urinary Hg levels	Occupational elemental Hg exposure [65,67,68]
***MTIM***	rs2270837	Children (8–12 years of age)/Portugal	Impaired behavioral performance involving the domains of visual spatial acuity and learning & memory, with some additional impacts on attention and motor function (boys); learning & memory (girls)	Urinary Hg level	Postnatal inorganic Hg exposure [47,61]
***MT2A***	rs10636	Children (8–12 years of age)/Portugal	Modulation of adverse effects on neurobehavioral (attention, visual spatial acuity, learning & memory), and motor functions (boys)	Urinary Hg level	Postnatal inorganic Hg exposure [47,61]
		Children (8 years of age)/UK	Increased cognitive consequences in children at school age	Umbilical cord Hg levels	Prenatal MeHg exposure [39]
***PON1***	rs662	Children (8 years of age)/UK	Cognitive deficit, associated with total and performance IQ, involving children at school age	Umbilical cord Hg levels	Prenatal MeHg exposure [39]
***PGR***	rs1042838	Children (8 years of age)/UK	Cognitive deficit, associated with total and verbal IQ, involving children at school age	Umbilical cord Hg levels	Prenatal MeHg exposure [39]
***SLC6A4*** 44bp ***(5-HTTLPR)*** Ins/Del		Children (8–12 years of age)/Portugal	Compromised neurobehavioral test performance affecting atten-tional control, and learning & memory (boys)	Urinary Hg levels	Postnatal inorganic Hg exposure [61]
		Adult (Dental assistants)/USA	Deteriorated cognitive skills for prolonged attention-memory, and psychomotor skills for cognitive flexibility, manual coordination (male dentists); attention, working memory, and manual coordination (female dental assistants). Increased mood scales between the two gender groups	Urinary Hg levels	Occupational elemental Hg exposure [69,70]
***TF***	rs3811647	Children (8 years of age)/UK	Decreased Performance IQ among children at school age	Umbilical cord Hg levels	Prenatal MeHg exposure [39]
***ABCC1*** rs11075290		Infants (20 months of age)/Republic of Seychelles	Compromised neuro-developmental test performance, affecting both mental and psychomotor development	Maternal hair Hg levels	Prenatal MeHg exposure [41]

* rs number.

**Table 4:** Genetic polymorphisms modulating the human susceptibility to mercury-induced cardiovascular damages.

Gene	Variant	Study Population/Location	Probable Genetic Impact on Cardiovascular Status/Estimated Interactions	Mercury Form
***GCLM***	5′near gene (* rs4130397)	Matched case-control subjects (age range: 30–77 years)/Northern Sweden	Increased risk of first ever-myocardial infarction, in people without cardiovascular disease/Erythrocyte Hg levels	MeHg [37]
***MMP-2***	5′near gene (rs243865)	Exposed subjects, through fish intake (15–87 years of age)/Brazilian Amazon	Potential risk of clinically relevant events, involving acute myocardial infarction, unstable or stable angina, and hypertension/Plasma Hg levels	MeHg [83]
***MMP-9***	Promoter (CA)_n_ repeats (rs3222264)	Exposed subjects, through fish intake (15–87 years of age)/Brazilian Amazon	Increased susceptibility to rapid coronary artery disease progression, fatal cardiovascular events or hypertension/Plasma Hg levels	MeHg [82]
***NOS3***	Intron 4 a/b (27 bpVNTR)	Fish eating populations (>18 years old)/Amazon region, Brazil	Increased systolic and diastolic blood pressures, with probable predisposition to hypertension, thrombosis, vasospasm, and atherosclerosis/Plasma and blood nitrile levels Hg-related	MeHg [84] MeHg [85]

* rs number.
